# Effect of Tryptophan Dietary Content on Meagre, *Argyrosomus regius*, Juveniles Stress and Behavioral Response

**DOI:** 10.3390/ani13243762

**Published:** 2023-12-06

**Authors:** Ana Vasconcelos, Marta C. Soares, Marisa Barata, Ana Couto, Bárbara Teixeira, Laura Ribeiro, Pedro Pousão-Ferreira, Rogério Mendes, Margarida Saavedra

**Affiliations:** 1 Faculdade de Ciências da Universidade do Porto, 4169-007 Porto, Portugal; santosvasconcelos@gmail.com; 2 CIBIO—Centro de Investigação em Biodiversidade e Recursos Genéticos, InBIO Laboratório Associado, Universidade do Porto, 4169-007 Porto, Portugal; marta.soares@cibio.up.pt; 3 BIOPOLIS Program in Genomics, Biodiversity and Land Planning, CIBIO, 4485-661 Vairão, Portugal; 4 MARE—Centro de Ciências do Mar e do Ambiente, ARNET—Rede de Investigação Aquática, Departamento de Paisagem, Universidade de Évora, Ambiente e Ordenamento, 7004-516 Évora, Portugal; 5 Aquaculture Research Station of IPMA, Av. Do Parque Natural da Ria Formosa, 8700-194 Olhão, Portugallribeiro@ipma.pt (L.R.); pedro.pousao@ipma.pt (P.P.-F.); 6 Interdisciplinary Centre of Marine and Environmental Research (CIIMAR), University of Porto, 4050-208 Matosinhos, Portugal; acouto@fc.up.pt (A.C.); barbara.p.b.teixeira@gmail.com (B.T.); rogerio@ipma.pt (R.M.); 7 Portuguese Institute for the Sea and Atmosphere, I.P. (IPMA), Division of Aquaculture, Upgrading and Bioprospection, 1495-165 Lisbon, Portugal; 8 MARE—Marine and Environmental Sciences Centre & ARNET—Aquatic Research Network Associated Laboratory, NOVA School of Science and Technology, NOVA University of Lisbon, 2829-516 Caparica, Portugal

**Keywords:** *Argyrosomus regius*, stress, tryptophan, behavior, cortisol

## Abstract

**Simple Summary:**

Fish welfare is an opportunity to improve the standards and quality of aquaculture products and is crucial to ensure the sustainability of the industry. Tryptophan is thought to mitigate fish response to stress. In this study, different dietary tryptophan contents were tested in meagre juveniles. The results suggest, particularly in a higher dosage (0.8%), a reduction of anxiety-like behavior in meagre exposure to acute stress. Although the remaining results showed mild effects of tryptophan dietary supplementation on meagre resilience to stress, it provides some clues as to the potential of this amino acid as a stress mitigator in aquaculture.

**Abstract:**

There are a high number of stressors present in aquaculture that can affect fish welfare and quality. One way of mitigating stress response is by increasing dietary tryptophan. In this study, three diets containing 0.5% (Tript1), 0.6% (Tript2), and 0.8% (Tript3) of tryptophan were tested in 32 g juvenile meagre for 56 days. At the end of the trial, survival, growth, and proximate composition were similar between treatments. Significant differences were found in the plasma parameters before and after a stress test consisting of 30 s of air exposure. Blood glucose levels were higher in the post-stress for all treatments (e.g., 63.9 and 76.7 mg/dL for Tript1 before and after the stress test), and the hemoglobin values were lower in the post-stress of Tript1 (1.9 g/dL compared to 3.0 and 2.4 g/dL for Tript2 and Tript3, respectively). In terms of behavior, three tests were carried out (novel tank diving and shoaling assays, and lateralization test), but no significant differences were found, except for the number of freezing episodes during the anxiety test (1.4 for Tript3 compared to 3.5 and 4.2 for the other treatments). This study suggests that supplementation with dietary tryptophan, particularly in higher dosage (0.8%), can reduce anxiety-like behavior in meagre exposure to acute stress (novel tank). Although the remaining results showed mild effects, they provide some clues as to the potential of this amino acid as a stress mitigator in aquaculture.

## 1. Introduction

Fish welfare is not only an ethical concern but also a means to improve the standards and quality of aquaculture products due to its implications, not only for production but for the sustainability of the industry [1] (FAO, 2019). Good husbandry conditions reduce stress in fish stemming from various internal and external factors [2,3], consequently lowering disease susceptibility, reducing medication needs, benefiting farmers, consumers, and the fish itself [4,5]. Therefore, efforts to minimize stress and disease incidence should be intensified to ensure a safer final product. Intensive aquaculture practices often prioritize maximizing production, impacting fish welfare due to heightened stressors, such as high densities [6]. These stressors can compromise fish welfare and increase the susceptibility to diseases. To counteract these effects, investment in stress resilience methodologies and immune system enhancement is essential. This not only improves animal welfare but also augments aquaculture sustainability [6].

Several methods have been employed to alleviate stress in farmed fish, with dietary supplementation being prevalent [6]. Enriching diets with specific amino acids involved in stress mechanisms, like tryptophan, has emerged as a potential strategy. Tryptophan is an essential amino acid involved in the synthesis of monoamines such as serotonin and melatonin [5,7]. Serotonin influences stress response and immune regulation, while melatonin is involved in immunity, stress response, and antioxidant capacity [7]. Tryptophan also inhibits superoxide production, scavenges free radicals, and mitigates pro-inflammatory cytokines production [4,7]. In rainbow trout (*Oncorhynchus mykiss*) and Senegalese Sole (*Solea senegalensis*), tryptophan maintained or reduced the basal levels of blood cortisol [8,9]. Consequently, supplementing diets with tryptophan may enhance stress resilience and fish welfare [4,7].

Fish behavior in aquaculture settings is a valuable indicator of animal response and stress, often overlooked [10]. Acknowledging the role of fish behavior can facilitate credible and simple health monitoring. Over the last decades, several tests, like the novel tank diving and shoaling tests, have been developed in laboratory conditions to evaluate anxiety levels and social behaviors in fish [11,12]. Additionally, lateralization analyses (described by Bisazza [13]) provide insights into cognitive functions related to habitat exploration, foraging, and escape from predators, which are crucial to meet the ecological and social demands involved in natural selection [14,15,16]. Despite their importance, these tests are not frequently used in aquaculture studies, offering a different perspective on fish stress response beyond physiological responses like cortisol levels. In the novel tank diving test, it is possible to compare several behavioral parameters to assess anxiety [11]. The shoaling test allows an understanding of fish’s individual response when near a small group of conspecific fish [12]. The lateralization test allows the assessment of brain lateralization that is involved in various routine actions, such as foraging [14,15,16].

This study aimed to evaluate the effect of dietary tryptophan supplementation on meagre behavior and physiological stress response. Meagre, chosen for its high commercial value and vulnerability to stress in intensive farming, was fed diets containing 0.5, 0.6, and 0.8% tryptophan to analyze meagre juveniles’ physiological and behavioral responses.

## 2. Material and Methods

This study was carried out at the Aquaculture Research Station of the Portuguese Institute for the Sea and Atmosphere (IPMA) between September and November 2021.

### 2.1. Husbandry and Experimental Set-Up

In this trial, 112 days old meagre with an initial weight of 32.6 ± 3.4 g and 14.4 ± 0.5 cm length were placed in nine 1500 L round fiberglass tanks, each containing 100 fish. The water circulated in an open circuit, passing through a cartridge filter before entering the tank. The water flow was approximately 15.9 L/min, oxygen saturation, and water temperature were 5.6 ± 0.2 mg/L and 21.4 ± 0.2 °C, respectively, while salinity was 38 ± 1 ppt. A 14 L/10 D photoperiod was maintained. 

Three diets with different tryptophan contents were tested in triplicates. The first diet (Tript1) contained 0.5% tryptophan content, approximating the estimated requirement for meagre of this age (preliminary trials suggested an optimal dietary tryptophan of approximately 0.5%). The other two diets (Tript2 and Tript3) were formulated with higher tryptophan levels (0.6 and 0.8, respectively).

The diets were formulated and produced by Sparos Lda (Olhão, Portugal). Powder ingredients were mixed in a double-helix mixer and ground twice in a micropulverizer hammer mill (SH1, Hosokawa-Alpine, Augsburg, Germany). Later, the oil fraction was added to the mixture, the diets were humidified, agglomerated via low-shear extrusion (Dominioni Group, Lurate Caccivio, Italy), and then dried in a convection oven (OP 750-UF, LTE Scientifics, Greenfield, UK) for 4 h at 60 °C. The diets were later crumbled (Neuero Farm, Melle, Germany) and sieved to 3 and 4 mm. The formulation of the three diets is presented in Table 1. Fish were hand-fed the diets ad libitum at 9 am, 11:30 am, 2 pm and 4:30 pm. The amount of feed given was quantified daily. The experimental trial lasted 56 days.

### 2.2. Sampling and Biochemical Analysis

Before the start of the experimental trial, 60 fish were individually weighed and measured. From this group, 10 fish were randomly selected for fish proximate composition analysis. The initial biomass of each tank was determined by weighing all fish grouped in pools of 10. In the mid-trial sampling, 20 fish per tank (60 per treatment) were collected to assess growth based on wet weight and length. At the end of the experiment, the remaining fish were again weighed in groups to establish the final biomass. Additionally, 61 fish per tank were individually weighed and measured. To analyze the fish’s final proximate composition, five fish per tank were collected (15 per treatment), and their samples were frozen at −20 °C.

Fish sampled for weight and length were sedated with 100 ppm 2-phenoxyethanol [17], while those collected for proximal composition and blood tests were euthanized using a higher concentration of phenoxyethanol. All handling of fish was carried out by trained scientists following category B FELASA recommendations and adhered to the European guidelines for the protection of animals used for scientific purposes (Directive 2010/63/UE of the European Parliament and of the European Union Council).

### 2.3. Chemical Analysis of Fish and Feed

Chemical analysis of the diets and carcasses was carried out following the methods of the Official Association of Chemical Analysts [18] and was run in duplicates. All the collected fish and feed were grounded prior to the analysis. To determine the dry matter, the samples were dried at 105 °C for 24 h. The ash content was obtained by incinerating the previous samples in a muffle furnace at 450 °C for 16 h. The lipid content was determined using the Soxhlet modified method (1879). The energetic value was determined using direct combustion in an adiabatic pump calorimeter (PARR Instruments, Moline, IL, USA; PARR model 1261). Fish protein content was determined using the Kjedahl-modified method (1883).

#### 2.3.1. Amino Acids Profile

##### Hydrolysis

The amino acid profiles of the three diets were determined according to the hydrolysis methods described in AOAC [19,20].

For the acidic hydrolysis, ca. 20 mg of the sample was hydrolyzed with 3 mL of 6 N HCl with 0.1% phenol, as described in Saavedra et al. [21].

For the alkaline hydrolysis, ca. 80 mg of sample was placed in 10 mL Nalgene Oak Ridge Teflon Fep tubes, with 3 mL of 4.2 N NaOH (deaerated by bubbling with N_2_ for 10 min) and one drop of 1-octanol. The hydrolysis was performed under inert conditions at 110–115 °C for 20 h, and then the samples were neutralized with HCl (4.2 N, 3.5 mL) and quantitatively transferred into 20 mL volumetric flasks with ultrapure water.

Norvaline and sarcosine were added to samples (final concentration 500 pmol/μL) before both types of hydrolysis and used as internal standards. Samples were filtered (0.2 m pore size) and stored at −80 °C until amino acid separation. All samples were analyzed in triplicate.

##### HPLC Analysis

The chromatographic conditions used were in accordance with the Agilent method [22] (Henderson et al., 2000) and amino acids separation was performed by high-performance liquid chromatography (Agilent 1100 HPLC, Agilent Technologies, Palo Alto, CA, USA) in a Phenomenex Gemini ODS C18 guard column (4 mm × 3 mm), and a Phenomenex Gemini ODS C18 110 Å column (4.6 mm × 150 mm, 5 μm) (Phenomenex Inc., Torrence, CA, USA) and detection wavelengths set by fluorescence (340/450 nm and 266/305 nm).

Amino acids identification and quantification were assessed by comparison to the retention times and peak areas of standard amino acids (Sigma, St. Louis, MO, USA) within the range 9–900 pmol/μL (R^2^ = 0.9999) with the software Agilent ChemStation for LC (Agilent Technologies, Palo Alto, CA, USA). All determinations were carried out in triplicate (repeatability 0.28–2.6% RSD; recovery 93–110%). Cysteine was not determined due to its destruction during acid hydrolysis.

### 2.4. Blood and Plasma Analysis

Blood was collected from 8 fish per tank (4 pre-stress and 4 post-stress) and analyzed individually. Between the stress test (30 s exposed to air) and the blood collection, an acclimation time of 30 min was given to the fish.

#### 2.4.1. Hematological Analysis

Blood aliquots were placed into a micro-hematocrit capillary tube (75 mm Super Rior, Lauda-Königshofen, Germany) and then spun in a micro-hematocrit centrifuge (EBA 21 Hettich, Tuttlingen, Germany) at 10,000× *g* for 5 min for hematocrit determination. For the hemoglobin analysis, a clinical analysis kit (ref. 1001239 SPINREACT) was used. After reagent and sample preparation, the absorbance was read at 540 nm in the microplate reader.

#### 2.4.2. Plasmatic Parameters

First, blood was centrifuged at 2500× *g* for 10 min to obtain plasma and stored at −80 °C until further analysis. Cortisol was determined using a cortisol ELISA kit test (RE52611, IBL International, Hamburg, Germany). Plasma glucose and lactate were analyzed using a clinical diagnostic kit from QCA (ref. 998660) and SPINREACT (ref. 1001330), respectively, based on the following reactions: glucose was based on the reaction of glucose oxidase and peroxidase, while lactate analysis was based on the reaction of lactate oxidase and peroxidase. Absorbance was read in the microplate reader at 340 nm and at 505 nm for glucose and lactate, respectively. Glucose and lactate were determined using analytical duplicates of each individual sample and analyzed using a colorimetric reaction on a microplate reader (Thermo Scientific, Waltham, MA, USA). For cortisol, two analytical samples were done.

### 2.5. Stress and Behavioral Tests

To assess varying levels of stress in fish behavior, three tests were carried out: the novel tank diving assay (anxiety assay, [11]), the shoaling assay [12], and the lateralization assay [13], with slight modifications made to the first two tests. These assessments are not often applied in aquaculture, particularly in nutrition trials. Each test involved evaluating four fish per tank (12 per treatment) that had fasted for 12 h. To minimize the metabolic variability observed throughout the day, treatments were evaluated alternately. Fish were given a five-minute acclimatization period in the test tank before the trial started. 

#### 2.5.1. Novel Tank Diving and Shoaling Assays

**Figure 1:**
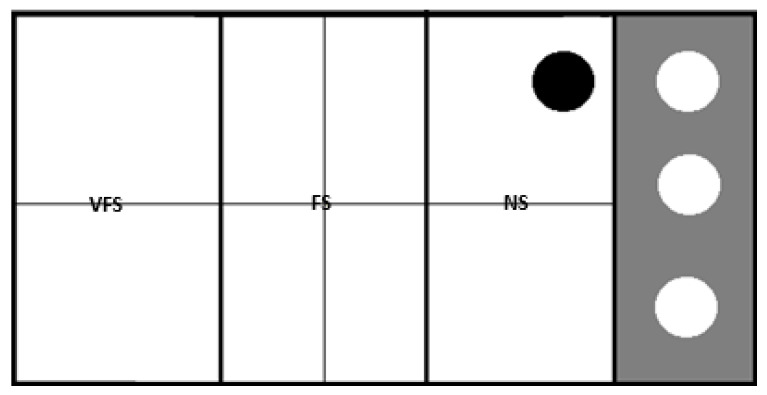
Schematic of the tank seen from above. Thicker lines—division of the three zones of the socialization test (NS, FS, VFS); thinner lines—division of the four sectors of the anxiety test (up/down, left/right); black circle represents the test individual, and white circles represent the shoal. VFS, very far from shoal; FS, far from shoal; NS, near shoal.

The anxiety assay and the shoaling assay took place in a novel tank with 240 L capacity (120 cm × 72 cm × 36 cm) (Figure 1) [11,12]. Fish behavior was recorded using two cameras, one located above the water level and another underwater, to allow better observation of the fish movements. Later, recorded images were reviewed for behavior analysis. To carry the anxiety assay, the tank’s right end was sectioned off with a transparent acrylic plate housing a group of six conspecific fish, while the tested fish occupied the remaining area. To prevent visual contact between the tested fish and the shoal, the acrylic was initially covered with an opaque plate until the trial began. For the anxiety assay, the tank was divided into four sectors (two upper and lower, and two left and right), using two lines. For the shoaling assay, the bottom of the tank was marked with two lines, creating three zones: near the shoal (NS), far from the shoal (FS), and very far from the shoal (VFS). Both tests had a duration of five minutes.

In the anxiety assay, the number of vertical and horizontal transitions were counted, as well as the time spent in the upper half of the tank and the number and time of freezing periods (time when the fish did not move). This test was carried out with the opaque plate on to avoid the interaction between the tested fish and the shoal, but once the anxiety assay was over, the opaque acrylic plate was removed, allowing the fish to have a full view of the shoal, and the start of the shoaling assay. In this trial, the time before the first approach to the shoal, the total time near and away from the shoal, the number of transitions towards or away from the shoal, the number of touches on the acrylic, and the number and time of freezing were quantified. 

#### 2.5.2. Lateralization Test

**Figure 2:**
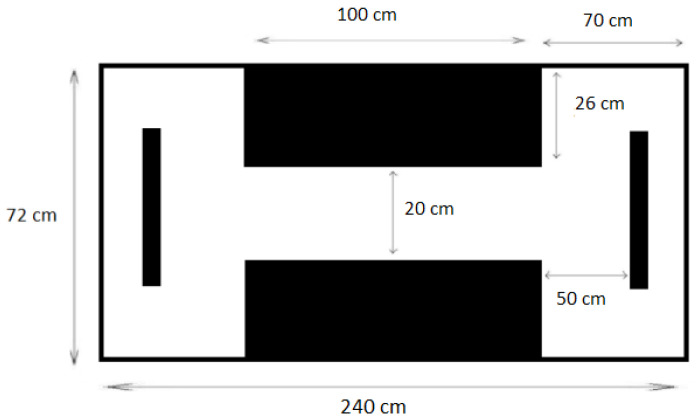
Schematic of the tank for the lateralization test (top view).

The lateralization assay was carried out in a novel tank with 480 L capacity (240 cm × 72 cm × 36 cm) featuring a maze and two barriers at opposite ends (Figure 2) [13]. Fish were introduced into the tank and placed in the center of the corridor, which remained closed with two grids during the acclimation time (5 min). Once the acclimation was over, the fish were allowed to swim freely, prompted by gentle water movement created using a fishing net, thus facing the obstacle and needing to choose between left and right.

Following the initial choice, each fish was required to return to the corridor and proceed to the opposite end, making a new decision. The test consisted of recording ten runs per fish. Subsequently, the relative index (LR) for each fish was calculated according to Bisazza et al. [13]. Values close to 100 indicated a preference for turning right in most of the 10 runs, values close to −100 indicated a left-turn preference, while values near zero indicated a similar preference between left and right.

### 2.6. Statistical Analyses

The presence of significant differences (*p* < 0.05) in plasma parameters before and after the stress test was analyzed using a two-way ANOVA. Cortisol values were compared using a two-way ANOVA, considering dietary tryptophan content and stress conditions as fixed factors. To comply with normality assumptions, a logarithmic transformation was performed in cortisol values. *t*-tests were used to identify post hoc differences between pre and post-test cortisol levels within each treatment. For proximate composition, growth (except final length, initial biomass, and survival), novel tank diving assay (except erratic movements), and shoaling assay (except latency time), one-way ANOVAs were used. For the parameters that did not satisfy the ANOVA assumptions, such as lactate, final length, initial biomass, survival, erratic movements and latency time, a non-parametric analysis (Kruskal–Wallis) was used. For the lateralization assay, chi square for independent samples was used. The specific growth rate (*SGR*) was calculated as $SGR=\frac{\ln DWf-\ln DWi}{t}\times 100$, where *DWf* and *DWi* are the final (*f*) and initial (*i*) dry weights (*DW*), respectively, and *t* the trial duration in days. Protein efficiency ratio (PER) was calculated as $PER=\frac{BIOf-BIOi}{Protein\;intake}$, where *BIOf* and *BIOi* are the final (*f*) and initial (*i*) biomass (*BIO*), respectively. The protein intake was calculated as Feed intake×Protein percentage. The feed conversion ratio (*FCR*) was calculated as $FCR=\frac{Feed\;intake}{weight\;gain}$.

The relative index (*LR*) was calculated as $LR=\frac{turns\;to\;the\;right-turns\;to\;the\;left}{turns\;to\;the\;right+turns\;to\;the\;left}\times 100$.

## 3. Results

### 3.1. Amino Acid Diet Composition

The analysis of the three diets confirmed that the diets had three significantly different tryptophan contents (Table 2). Tript1 had a 0.5% tryptophan, Tript2 had 0.6%, and Tript3 had the highest content, 0.8%. When the AA profile of diets was analyzed, differences were observed in the content of aspartate, serine, threonine, and isoleucine, especially between diets Tript1 and Tript2 (Table 2).

### 3.2. Survival and Growth

Survival varied between 94% and 96%, and the values were not significantly different between treatments. At the end of the trial, no significant differences in fish length and weight between treatments were observed. The same was observed for parameters such as biomass, SGR, FCR, and PER, which did not show any significant differences between treatments (Table 3). Diet composition did not seem to affect fish, with proximate composition being approximately 15% protein and 4.7% lipid content (Table 4).

### 3.3. Blood Analysis

The hematocrit did not show significant differences between treatments or fish subjected/not subjected to a stress test. However, in meagre fed the Tript1 diet, hemoglobin decreased after fish were submitted to the stress test. In the other diets with higher tryptophan, hemoglobin levels were not affected by the stress test (Table 5).

For blood glucose levels, no significant differences were observed between treatments before the stress test. After the stress test, an increase in the glucose levels was observed in all treatments, being significantly higher in Tript1. Additionally, the glucose levels in the blood were significantly lower in Tript3 compared to Tript1, after the stress test (Table 5). The concentration of lactate was not affected by the tryptophan level but was significantly affected by the stress test, with the levels decreasing after the stress test (Table 5).

**Figure 3:**
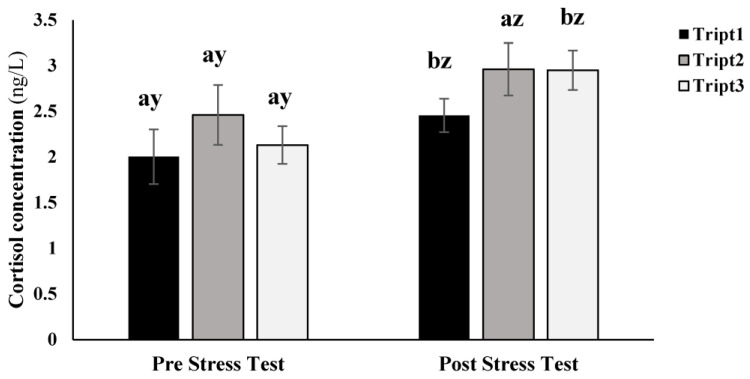
Cortisol blood concentration in the plasma before and after fish were submitted to a stress test consisting of 30 s outside water. Treatments tested three diets with 0.5% (Tript1), 0.6% (Tript2), and 0.8% (Tript 3) of tryptophan in meagre juveniles. Different letters correspond to significant differences between pre and poststress test for the same treatment (a/b) and between pre and poststress test for all treatments (y/z) for *p* < 0.05.

The cortisol levels in the plasma did not seem to be affected by the diet but were affected by the stress test. Fish from Tript1 and Tript3 showed a significant increase in the concentration of cortisol after being subjected to the stress test. In Tript2, no significant differences were found before and after the stress test (Figure 3).

### 3.4. Stress and Behavioral Tests

To evaluate the effect of dietary tryptophan content, three tests were carried out: the novel tank diving assay (anxiety test), the shoaling assay, and the lateralization assay.

#### 3.4.1. Novel Tank Diving Assay

In this assay, fish from Tript3 had more transitions between sectors and spent more time in the upper part of the tank, while fish from Tript1 and Tript2 had a similar number of transitions and meagre from treatment Tript2 spent less time in the upper half of the tank (Table 6). Fish from Tript1 and Tript2 had more episodes and longer periods of freezing (Table 6). Nevertheless, these differences were not significant.

#### 3.4.2. Shoaling Assay

In this assay, meagre from Tript2 took less time to approach the shoal, stayed next to the shoal for a longer period, and had fewer transitions and a lower number and duration of freezing episodes. On the other hand, fish from Tript3 took more time to approach the shoal, touched the acrylic more often, and had longer episodes of freezing (similar to Tript1). Meagre from Tript1 stayed next to the shoal for a shorter period, had more transitions, touched the acrylic less often, and had more episodes of freezing. However, these differences between treatments were not significantly different (Table 7).

#### 3.4.3. Lateralization Assay

The lateralization assay evaluated the fish’s choice of sides when it ran into an obstacle. Fish from Tript2 turned left most of the time. On the contrary, fish from treatment Tript1 chose right more. Nevertheless, there was a high variation between individuals, and no significant differences were found between treatments. For this, the relative lateralization index was used. The lateralization index was 18.3 for Tript1, −11.7 for Tript2 and 0.0 for Tript3 (Table 8).

## 4. Discussion

In various countries, concerns regarding fish welfare in aquaculture are increasing, leading to intensified efforts to mitigate fish stress during the rearing process. Consequently, research is now focused on diverse strategies aimed at enhancing fish welfare. This endeavor seeks to bolster consumer trust in the industry and increase the value of aquaculture products. This study investigates the potential of dietary tryptophan supplementation- an amino acid implicated in fish stress response- to bolster resilience against stressors prevalent in daily aquaculture activities. Preliminary nutritional trials suggested an optimal dietary tryptophan level for meagre juveniles around 0.5%, corresponding to the tryptophan content in the Tript1 diet. Additionally, two other diets containing higher tryptophan contents were evaluated. 

The survival rate obtained in this study ranged from 94 to 96%, aligning with expected levels for this species at this developmental stage [23,24,25,26]. Mortality primarily stemmed from fish jumping out of the tank, a behavior often linked to stress episodes. This emphasizes the need to mitigate stress in this species.

Regarding growth parameters, no significant differences were found in the midtrial or at the end of the experiment. This can be attributed to the diets’ high similarity in composition and the absence of tryptophan deficiency, thus not limiting protein synthesis. These findings align with the SGR, FCR, and PER values, which also displayed no significant differences among treatments. The SGR and FCR values obtained in this study are in the same range as those reported by Herrera et al. [27] and Saavedra et al. [24] using juveniles from the same species.

Likewise, fish proximate composition remained consistent across treatments. These outcomes agree with similar findings involving meagre juveniles [24,25]. Studies examining tryptophan supplementation have reported varied effects. For instance, Hoseini et al. [28] investigated different levels of tryptophan in rainbow trout juveniles (*Oncorhynchus mykiss*) diets, observing no significant differences in carcass composition. Conversely, Sharf and Khan [29] observed a significant increase in lipid and protein content when testing diets with up to 0.47% tryptophan fed to *Channa punctatus*.

Hematological parameters are good indicators of nutritional status [30] and are important in evaluating physiological and pathological changes in fish [24,31]. Tryptophan has been shown to mitigate fish response to stress in several aquaculture species, such as carps [32,33], totoaba [5] and striped bass [34]. In the present study, tryptophan supplementation did not significantly affect meagre response to the stress test, resulting in significant increases in cortisol levels after the stress test, except for fish fed 0.6% tryptophan, which did not exhibit a significant rise in plasma cortisol levels. However, due to considerable variability, confirming increased fish resilience is challenging. Moreover, cortisol levels after the stress test did not decrease compared to those from the other treatments. Similarly, glucose levels increased after the stress test, especially in fish fed 0.5% tryptophan, potentially associated with heightened energy resources catabolism. In the primary stress response, cortisol release triggers a secondary response, leading to increased glucose levels and its transport to energy-demanding tissues for restoration [5,35,36]. This is consistent with the results obtained for the treatment with lower tryptophan content and coincident with those reported by Saavedra et al. [24] for meagre, Abdel-Tawwab [35] for Nile tilapia and Hoseini et al. [37] for sturgeon. However, it was anticipated that a higher dietary tryptophan content could decrease blood glucose levels, as described by Herrera et al. [27], or at least maintain glucose levels before and after the stress test, as observed by Hoseini et al. [28]. The latter was witnessed in treatments with 0.6 and 0.8% of tryptophan.

Contrarily to glucose, lactate results are unexpected and complex to explain. Normally, exposure to air increases muscle glucose anaerobic catabolism, elevating lactate production [36]. Yet, in this study, lactate concentration decreased after the stress test, contrary to Saavedra et al. [24], where meagre juveniles showed increased lactate levels under similar stress conditions. Moreover, the lactate values in this study were considerably lower than those reported by Saavedra et al. [24]. According to Monteiro et al. [36], this discrepancy could be related to the nutritional balance of the diets, as fish-fed balanced diets could maintain lower lactate levels in the blood. Further, it might also indicate that the tryptophan levels in the diets had the desired effect.

Regarding other blood parameters like hematocrit, no significant differences were obtained between treatments or due to the stress test, with values within the previously published range for meagre [24]. However, hemoglobin concentration was affected by the stress test in meagre fed a diet with 0.5% tryptophan, resulting in reduced levels post-test. Some studies have reported similar decreases in hemoglobin during stress. Kpundeh et al. [38] observed reduced hemoglobin values in tilapia juveniles (*Oreochromis niloticus*) subjected to stressors. In this study, hemoglobin reduction was specific to the 0.5% tryptophan treatment, implying the potential positive effects of tryptophan supplementation on this parameter and its impact on the endocrine response to stress [5].

It was deemed important to analyze fish behavioral response, an aspect seldom explored in aquaculture studies. The focus was on three important components: fish anxiety in exploring a new environment (novel tank), fish social response, and fish brain lateralization. In the anxiety test, significant differences were not found overall, except for the number of freezing episodes. According to Maulvault et al. [14] and Egan et al. [11], an increase in these episodes is associated with higher levels of stress-related hormones (e.g., cortisol) and anxiety in general. This study revealed a decrease in the number of freezing episodes in the treatment with the highest tryptophan content, suggesting that a higher dietary tryptophan might present an anxiolytic effect, potentially alleviating behavioral and endocrine effects of acute stress. These behaviors are typically characterized by higher cortisol levels in the blood and are influenced by individual fish traits [39] However, no other differences were observed in the other parameters analyzed, potentially due to high individual variability. Other studies [40] tested tryptophan administration through the water and did not observe differences in adult zebrafish behavior.

The shoaling test showed no differences between treatments. This outcome is unsurprising as meagre is a shoaling species, and it is expected for an individual to approach conspecifics within a few seconds. The waiting time until approaching the shoal was comparable among treatments (Tript1—6.4 s, Tript2—2.8 s, and Tript3—6.7 s), although high variability was noted in fish fed 0.8% tryptophan, which might have influenced the results. Maulvault et al. [14] observed significant differences in the time spent before the first approach to the shoal when individuals were exposed to venlafaxine via their feed. However, no significant differences were found in the time spent near the shoal, which suggests that individuals from different treatments had a similar eagerness to join the shoal, which is crucial for survival in nature.

Brain lateralization, driven by brain asymmetry, aids common animal behaviors like escaping from predators, offering a selective advantage [14]. In the lateralization assay, no significant differences were observed among the three tryptophan diets. The lateralization index (LR) in this study showed high variability, akin to Roche et al. [16], possibly masking potential differences between treatments.

## 5. Conclusions

In conclusion, this study adopted diverse approaches to meagre welfare, not solely relying on physiological parameters but also considering fish behavior, an uncommon approach in aquaculture studies. It suggests that dietary supplementation of tryptophan, particularly at a higher dosage (0.8% of tryptophan), can reduce anxiety-like behavior in meagre exposed to acute stress in a novel tank. Other results indicated mild effects of tryptophan dietary supplementation on meagre resilience to stress, potentially influenced by high variability in fish individual responses or by a nonexistent tryptophan depletion group. Nevertheless, this study provides insights into the potential of this amino acid as a stress mitigator in aquaculture.

## Figures and Tables

**Table 1:** Ingredients and proximate composition of the experimental diets. Tript 1—with 0.5% of dietary tryptophan; Tript 2—0.6%; Tript 3—0.8%.

Ingredients (%)	Tript1	Tript2	Tript3
Casein	5	5	5
Porcine gelatin	6	6	6
Soy protein concentrate	30	30	30
Pea protein concentrate	15	15	15
Wheat gluten	15	15	15
Potato starch	7	6.6	5.6
Fish oil	7	7	7
Rapeseed oil	7	7	7
Rapeseed lecithin	2	2	2
Vitamin and minerals	1	1	1
Vitamin C	0.1	0.1	0.1
Vitamin E	0.1	0.1	0.1
Betaine HCl	1	1	1
Antioxidant	0.3	0.3	0.3
Monoammonium phosphate	2	2	2
L-Lysine	0.1	0.1	0.1
L-Tryptophan	0.2	0.4	0.8
Composition (% DW)			
Protein	52.0	52.3	52.2
Lipids	16.0	17.5	16.0
Ash	7.6	7.4	7.8
Energy (KJ)	23.9	24.1	24.1

**Table 2:** Amino acid composition (g/100 g) of the diets. Tript1—with 0.5% of dietary tryptophan; Tript2—0.6%; Tript3—0.8%. Values are mean and standard error. Different bold letters represent significant differences for *p* < 0.05.

Amino Acids	Tript1	Tript2	Tript3
Aspartate	2.7 ± 0.0 ^**a**^	2.9 ± 0.0 ^**b**^	2.9 ± 0.0 ^**ab**^
Glutamate	8.1 ± 0.0	8.7 ± 0.0	8.6 ± 0.1
Serine	2.0 ± 0.0 ^**a**^	2.1 ± 0.0 ^**b**^	2.1 ± 0.0 ^**ab**^
Histidine	0.6 ± 0.0	0.7 ± 0.0	0.7 ± 0.0
Glycine	3.4 ± 0.0	3.4 ± 0.0	3.1 ± 0.0
Threonine	2.1 ± 0.0 ^**a**^	2.3 ± 0.0 ^**b**^	2.2 ± 0.0 ^**ab**^
Arginine	2.1 ± 0.0	2.3 ± 0.0	2.2 ± 0.0
Alanine	3.2 ± 0.0	3.4 ± 0.0	3.2 ± 0.0
Taurine	2.5 ± 0.0 ^**a**^	2.3 ± 0.0 ^**b**^	2.2 ± 0.0 ^**b**^
Tyrosine	1.4 ± 0.0	1.6 ± 0.0	1.5 ± 0.0
Valine	1.6 ± 0.0 ^**a**^	1.7 ± 0.0 ^**b**^	1.6 ± 0.0 ^**a**^
Methionine	0.7 ± 0.0	0.7 ± 0.0	0.7 ± 0.0
Tryptophan	0.5 ± 0.0 ^**a**^	0.6 ± 0.0 ^**b**^	0.8 ± 0.0 ^**c**^
Phenylalanine	1.9 ± 0.0 ^**a**^	2.0 ± 0.0 ^**b**^	2.0 ± 0.0 ^**ab**^
Isoleucine	1.3 ± 0.0 ^**a**^	1.4 ± 0.0 ^**b**^	1.3 ± 0.0 ^**ab**^
Leucine	4.0 ± 0.0	4.3 ± 0.0	4.2 ± 0.0
Lysine	0.4 ± 0.0	0.3 ± 0.0	0.3 ± 0.0
Hydroxyproline	0.9 ± 0.0 ^**a**^	1.0 ± 0.0 ^**b**^	0.9 ± 0.0 ^**a**^
Proline	4.0 ± 0.0 ^**a**^	4.4 ± 0.0 ^**b**^	4.1 ± 0.0 ^**ab**^

**Table 3:** Meagre juvenile biometry in the beginning (112 days), midterm (146 days), and end (174 days) of the experimental trial, which tested three diets with 0.5%, 0.6%, and 0.8% of tryptophan. SGR—specific growth rate; FCR—feed conversion rate; ER—protein efficiency rate. Tript1—with 0.5% of dietary tryptophan; Tript2—0.6%; Tript3—0.8%.Values are mean and standard error.

Treatment	Tript1	Tript2	Tript3
Survival (%)	95.3 ± 0.6	95.7 ± 0.2	94.3 ± 1.5
Weight (g)			
112 days	32.6 ± 0.1	32.6 ± 0.1	32.6 ± 0.1
146 days	47.3 ± 0.6	50.4 ± 0.4	48.9 ± 0.9
174 days	61.6 ± 1.1	63.5 ± 0.3	64.3 ± 0.6
Length (cm)			
112 days	14.4 ± 0.0	14.4 ± 0.0	14.4 ± 0.0
146 days	16.8 ± 0.0	17.1 ± 0.1	17.0 ± 0.0
174 days	18.5 ± 0.1	18.6 ± 0.0	18.5 ± 0.0
Biomass (g)			
112 days	3325.4 ± 0.8	3320.2 ± 0.6	3314.7 ± 2.9
146 days	946 ± 11.0	1008.7 ± 8.5	975.7 ± 6.0
174 days	5878.6 ± 128.7	6077.9 ± 32.6	6071.2 ± 126.6
SGR	1.1 ± 0.03	1.2 ± 0.01	1.2 ± 0.02
FCR	1.2 ± 0.03	1.1 ± 0.01	1.1 ± 0.03
PER	1.6 ± 0.04	1.7 ± 0.01	1.7 ± 0.05

**Table 4:** Proximate composition in a fresh matter of meagre juveniles at the end of the trial fed three diets with 0.5%, 0.6%, and 0.8% of tryptophan. Tript1—with 0.5% of dietary tryptophan; Tript2—0.6%; Tript3—0.8%.Values are mean and standard error.

Treatment	Tript1	Tript2	Tript3
Crude protein	15.7 ± 0.0	15.4 ± 0.2	15.4 ± 0.1
Crude fat	4.9 ± 0.1	4.7 ± 0.2	4.6 ± 0.1
% Dry matter	24.9 ± 0.0	25.0 ± 0.0	24.8 ± 0.0
% Ashes	3.8 ± 0.0	4.0 ± 0.0	3.6 ± 0.0
Energy kJ/g (FM)	5.6 ± 0.0	5.6 ± 0.0	5.6 ± 0.0

**Table 5:** Blood parameters in meagre juveniles before and after being submitted to a stress test. Tript1—with 0.5% of dietary tryptophan; Tript2—0.6%; Tript3—0.8%. Values are mean and SE. Different letters represent significant differences for *p* < 0.05. HTC, hematocrit; Hgb, hemoglobin; Glu, glucose.

Treatment	Tript1		Tript2		Tript3	
Stress Test	Pre	Post	Pre	Post	Pre	Post
HTC (%)	20.4 ± 0.2	20.3 ± 0.1	19.8 ± 0.2	20.3 ± 0.2	20.3 ± 0.2	18.8 ± 0.1
Hgb (g/dL)	3.0 ± 0.1 ^a^	1.9 ± 0.0 ^b^	2.7 ± 0.1 ^a^	3.0 ± 0.1 ^a^	2.9 ± 0.1 ^a^	2.4 ± 0.1 ^ab^
Glu (mg/dL)	63.9 ± 1.0 ^a^	76.7 ± 0.7 ^b^	63.4 ± 0.8 ^a^	74.1 ± 1.0 ^bc^	63.5 ± 0.8 ^a^	73.2 ± 0.7 ^c^
Lactate (mg/dL)	3.5 ± 0.1 ^a^	1.8 ± 0.0 ^b^	3.1 ± 0.1 ^a^	1.6 ± 0.0 ^b^	3.1 ± 0.1 ^a^	1.6 ± 0.0 ^b^

**Table 6:** Results were obtained in the novel tank diving assay of meagre juveniles fed with three diets with 0.5%, 0.6%, and 0.8% of tryptophan. The freezing percentage corresponds to the time fish had no movement of the 5-min test. Tript1—with 0.5% of dietary tryptophan; Tript2—0.6%; Tript3—0.8%. Values are mean and standard error. Different letters correspond to significant differences for *p* < 0.5.

Treatment	Tript1	Tript2	Tript3
Total transitions	53.9 ± 2.5	57.0 ± 3.0	72.7 ± 3.8
Time on the upper half (%)	39.6 ± 2.7	31.5 ± 2.0	42.1 ± 2.8
Number of freezing	4.2 ± 0.3 ^a^	3.5 ± 0.2 ^ab^	1.4 ± 0.2 ^b^
Freezing time (%)	20.3 ± 3.5	29.4 ± 0.8	16.8 ± 5.2

**Table 7:** Results were obtained in the shoaling assay (5 min) of meagre juveniles fed with three diets with 0.5%, 0.6%, and 0.8% of tryptophan. Values are mean and standard error.

Treatment	Tript1	Tript2	Tript3
Time prior to 1st approach/300 s	0.4 ± 0.23	0.2 ± 0.2	2.2 ± 0.8
Time next to the shoal (%)	81.1 ± 3.5	86.5 ± 3.6	86.2 ± 2.6
Total transitions	17.0 ± 2.9	14.1 ± 3.7	15.7 ± 4.1
Touches on the acrylic/300 s	18.6 ± 2.6	23.8 ± 3.1	24.0 ± 1.4
Number of freezing/300 s	5.4 ± 1.6	3.8 ± 1.1	4.1 ± 0.9

**Table 8:** Results were obtained in the lateralization assay of meagre juveniles fed with three diets with 0.5%, 0.6%, and 0.8% of tryptophan. Values are mean and standard deviation.

Treatment	Tript1	Tript2	Tript3
Times choosing left	4.1 ± 0.2	5.6 ± 0.2	5.0 ± 0.2
Relative lateralization index	18.3 ± 3.1	−11.7 ± 4.2	0.0 ± 3.5

## Data Availability

Data used in the manuscript for tables and figures are available upon request.
